# Multimorbidity, clinical decision making and health care delivery in New Zealand Primary care: a qualitative study

**DOI:** 10.1186/s12875-017-0622-4

**Published:** 2017-04-05

**Authors:** Tim Stokes, Emma Tumilty, Fiona Doolan-Noble, Robin Gauld

**Affiliations:** 1 Department of General Practice and Rural Health, Dunedin School of Medicine, University of Otago, PO Box 56, Dunedin, 9054 New Zealand; 2 Otago Business School, University of Otago, Dunedin, New Zealand

**Keywords:** Multimorbidity, Primary care, General practice, Health services, Decision making

## Abstract

**Background:**

Multimorbidity is a major issue for primary care. We aimed to explore primary care professionals’ accounts of managing multimorbidity and its impact on clinical decision making and regional health care delivery.

**Methods:**

Qualitative interviews with 12 General Practitioners and 4 Primary Care Nurses in New Zealand’s Otago region. Thematic analysis was conducted using the constant comparative method.

**Results:**

Primary care professionals encountered challenges in providing care to patients with multimorbidity with respect to both clinical decision making and health care delivery. Clinical decision making occurred in time-limited consultations where the challenges of complexity and inadequacy of single disease guidelines were managed through the use of “satisficing” (care deemed satisfactory and sufficient for a given patient) and sequential consultations utilising relational continuity of care. The New Zealand primary care co-payment funding model was seen as a barrier to the delivery of care as it discourages sequential consultations, a problem only partially addressed through the use of the additional capitation based funding stream of *Care Plus*. Fragmentation of care also occurred within general practice and across the primary/secondary care interface.

**Conclusions:**

These findings highlight specific New Zealand barriers to the delivery of primary care to patients living with multimorbidity. There is a need to develop, implement and nationally evaluate a revised version of *Care Plus* that takes account of these barriers.

**Electronic supplementary material:**

The online version of this article (doi:10.1186/s12875-017-0622-4) contains supplementary material, which is available to authorized users.

## Background

Multimorbidity (the presence of two or more chronic conditions in a single patient) [1] is one of the biggest challenges facing health systems internationally as multiple disease care, not single disease care, becomes the norm in an aging society [2, 3] Multimorbidity is a major issue in primary care [4]. Recent epidemiological studies of chronic disease show that multimorbidity is the norm for people aged over 65 [5]. For example, a large Scottish primary care cross sectional study found that 23% of all patients were multimorbid with a prevalence rising to 65% in the 65-84 age group [6]. Multimorbidity leads to poorer health outcomes: it is associated with high mortality, reduced functional status and quality of life and increased use of inpatient and ambulatory health care [7, 8]. Research has identified that certain health care delivery interventions (e.g., enhanced multidisciplinary team care with structured visits) may improve health outcomes for older people with multimorbidity [9].

Chronic disease management is predominantly delivered using a traditional single disease model which means people with multimorbidity receive fragmented, inefficient and duplicate health care delivery [7, 8, 10]. There is therefore a need to develop new models of care to ensure integrated care for people with multimorbidity which is both effective and financially sustainable [3, 11]. A strong primary care health system is crucial to such integration and it is a central tenet of general practice care that it should be patient, not disease centred, and promote shared decision making [10, 12]. A recent qualitative synthesis of published studies exploring general practitioners (GPs’) management of patients with multimorbidity found GPs faced a number of significant challenges delivering care to this group of which the disorganisation and fragmentation of health care, challenges in delivering patient centred care, inadequacy of clinical guidelines and barriers to shared decision making were key themes [13].

Multimorbidity research to date has largely been conducted in health systems outside of New Zealand (NZ). NZ has a strong first contact primary care health system (see Table 1). GPs are predominantly independent, self-employed providers with 50% of their funding coming from a capitated government-determined subsidy, paid through Primary Health Organisations (PHOs). The remainder of their funding comes from individual patient co-payments, which are set by each GP practice. The average patient co-payment for a GP consultation for an adult ranges from NZD15 to NZD45 (USD10–USD31) [14]. There is also a lower patient co-payment in general practices with “high needs”(>50% Māori; Pacific; lowest socioeconomic status) [15]. As with other similar health systems, the NZ health care system is struggling to cope with the rise in long term conditions as well as the increasing prevalence of multimorbidity [11, 16].

**Table 1:** The New Zealand Health System

New Zealand’s 1938 Social Security Act was the world’s first attempt to create a “national health service”, but doctor resistance meant this was never achieved. A series of policy compromises mean that, today, public hospitals salary all staff and are free of patient charges. General Practitioners mostly practice privately and act as gatekeepers. They receive considerable government subsidies but charge most patients a fee per consultation, creating an access barrier [37]. There is a strong tradition of family practice and focus on primary care within the health system. Yet, the arrangements set down in the post-1938 compromise mean GPs and public hospitals work largely separately from one another. Government contributes around 80% of total health expenditure. Around 40% of public hospital specialists have a separate private practice. The parallel private system means patients of better means are able to circumvent public hospital waiting times when referred by their GP, or to access treatments considered to be lower priority in the constrained public sector [49].	

In 2004 the NZ Ministry of Health introduced *Care Plus*, an additional capitation based funding stream for primary care, which aims to “improve chronic care management, reduce inequalities, improve primary care teamwork and reduce the cost of services for high-need patients” [17–19]. *Care Plus* subsidises four extended consultations annually and its eligibility criteria [20] are presented in Table 2. A related payment stream is the provision of a High Use Health Card (HUHC), for which a patient must have received at least 12 health practitioner consultations within the last 12 months for a particular ongoing medical condition(s). *Care Plus* differs from the HUHC, however, in that it is intended to coordinate “a comprehensive approach to improve outcomes for people with chronic conditions, including lower cost access; whereas the HUHC is a subsidy approach tied to GP visits” [20]. The national *Care Plus* initiative has been complemented by regional health service initiatives. For example, in the Southern Health Region of NZ’s South Island (Otago and Southland), the District Health Board (DHB) and PHO are working to further service integration through Alliance South [21], which is a contractual alliance between the two organisations aimed at improving care coordination and integration. Alliance South is developing a strategic health services plan in which better chronic disease management is a key priority [22]. If local health care services are to be redesigned to better meet the needs of patients living with multimorbidity then it is critical that this is informed by an understanding of the barriers and enablers to primary care’s role in the management of such patients.

**Table 2:** Eligibility criteria for Care Plus [20]

A general practice that is part of a PHO can enrol a patient in *Care Plus* if they are assessed by a doctor or nurse at the general practice as:
• being able to benefit from intensive clinical management in primary health care (at least 2 h of care from 1 or more members of the primary health care team over the following 6 months), and
• having 2 or more chronic health conditions, as long as each condition is one that:
• is a significant disability or has a significant burden of morbidity; and
• creates a significant cost to the health system; and
• has agreed and objective diagnostic criteria; and
• requires continuity of care and where a primary health care team approach has an important role in management; or
• requiring intensive clinical care because they:
• have a terminal illness (defined as someone who has advanced, progressive disease whose death is likely within 12 months); or
• have had 2 acute medical or mental health-related hospital admissions in the past 12 months (excluding surgical admissions); or
• have had 6 first-level service or similar primary health care visits in the past 12 months (including emergency department visits); or
• are on active review for elective services.

In this context, this study aimed to explore primary care professionals’ - GP and primary care nurses (PCNs) (practice nurses and nurse practitioners) - accounts of managing multimorbidity in one NZ health region and the impact of this on clinical decision making and health care delivery.

## Methods

### Design and sampling

Semi-structured interviews were conducted between May and November 2015 with GPs and PCNs working in general practices throughout the Otago region. Otago, in the south of NZ's South Island, is the second-largest NZ region in terms of land area. It has a population of 202,467 (2013 NZ Census), being 4.8% of NZ’s population [23]. The city of Dunedin on the east coast is the regional centre. Otago overall has a much lower proportion of Māori (2.4%) than NZ as a whole (14.9%) [23] although it has areas with high Māori and Pasifika populations (e.g., South Dunedin). GPs and PCNs were sampled purposively in order to construct a maximum variation sample that reflected practice characteristics - such as number of GPs, level of deprivation and location (urban and rural) – that are related to practice organisation and chronic disease and multimorbidity prevalence [6].

### Data collection

The interviews used a topic guide based on a literature review and discussions within the research team. The topic guide (see Additional file 1) covered: a) the organisation of local health care services and possible funding models for people with single conditions and multimorbidity; b) clinical management by GPs and PCNs (this was explored through participants describing situations where care for multimorbid patients was considered to have been delivered both well and poorly). The topic guide was used flexibly to allow participants to construct their accounts in their own terms, and was revised and refined throughout the interviewing process to reflect themes emerging from the concurrent data analysis. All interviews were digitally-recorded and transcribed verbatim.

### Data analysis

A thematic analysis was conducted using the constant comparative method [24, 25]. A coding framework was developed from the initial interviews by ET, TS and FDN. Through an iterative process involving comparison across the transcripts, assisted by NVivo 10 qualitative analysis software, these descriptive codes were organized by ET into higher order thematic categories. TS and FDN independently assessed the plausibility and explanatory value of the categories against the transcripts, and also independently evaluated the assignment of a sample of the data to the categories. A reflexive diary of the analysis was maintained. This provided an ‘audit trail’ of the development of the framework and its categories and also promoted reflexive research practice. The consolidated criteria for reporting qualitative research (COREQ) (See Additional file 2) [26] were used to inform reporting of the findings.

## Results

Twelve GPs and four PCNs were interviewed. All participants were involved in delivering chronic disease/long term condition management. The characteristics of participants and their general practices are shown in Table 3. Participants showed wide variation in terms of their personal characteristics and practice demography.

**Table 3:** Characteristics of GP and Primary Care Nurse (PCN) participants (*N* = 16) and of general practice demographics (*N* = 15)

Category	Participants, *n* (%)
*GP and PCN participants*	*16 (100)*
Profession:	
General Practitioner (GP)	12 (75)
Primary care Nurse (PCN)^a^	4 (25)
Sex:	
Female	7 (44)
Male	9 (56)
Ethnicity:	
New Zealand European	11 (69)
Other (including: Asian, Other European, African, etc.)^b^	5 (31)
Years in clinical practice:	
0–5 years	1 (6)
6–10 years	3 (19)
11–20 years	3 (19)
20 years +	9 (56)
*General Practice demographics* ^c^	*15 (100)*
Practice Size:	
1–4 GPs	6 (40)
5–9 GPs	4 (27)
10+ GPs	5 (33)
Practice Location:	
Urban	9 (60)
Rural	6 (40)
Practice location New Zealand Deprivation Index	
1–3	4 (27)
4–7	10 (67)
8+	1 (6)

^a^Specific roles were: three nurses were based in general practice: nurse practitioner (1), practice nurse (2) and one was based in the relevant primary health organisation

^b^Non-New Zealand European categories were conflated to ensure participant anonymity

^c^One participant did not currently work in a general practice but within another local health organisation (primary health organisation)

We report here the themes that emerged from the interviews in terms of clinical decision making and health care delivery for patients with multimorbidity. Illustrative participant quotes are presented.

### Clinical decision making

#### Complexity

All participants reported that lack of time within the NZ 15 min general practice consultation “standard” appointment length was an issue in terms of not only addressing a multimorbid patient’s health needs, but also in communicating, prioritising, agreeing plans and endeavouring to get the patient engaged in self-management. Multimorbid patients were seen as complex:

*That's a multi-morbidity, I can think of where there's a complex array of medical conditions which prevents that person from being able to cope alone at home, bringing on confusion with the number of medications that the person is on, and so on and so forth. It's like a big waterfall. (Participant 1 GP)*

This complexity caused difficulty in managing care for patients with multimorbidity based on number of items on the patients’ agenda to be addressed in the time available:

*It’s like people come in with their shopping list and they want a repeat of their 15 different interacting medications for their 6 six different pathologies. (…) (Participant 6 GP)*

Further difficulty was experienced in trying to both negotiate with patients over their priorities and the ones the participants felt needed to be addressed that day. There was evidence of “safety netting” by participants, where GPs incorporate issues they felt needed attention in a consultation even if these were not a patient priority:

*I think often they've got their agenda of what they want to talk about. You've got your idea that, okay, you want your prescriptions, but I also have to check a number of other things. Trying to focus on what they've actually come in for, which may not be the most urgent thing but is obviously the thing that's worrying them the most, and picking at there's nothing particularly dangerous that you're missing like the ones who at the end of the consult say, "Oh by the way, I've been having chest pain for the last six weeks." (Participant 2 GP)*

While participants noted that the use of “catch up” time in the middle of their standard booked clinics allowed some flexibility to spend more time on individual patients this was constrained by financial cost (see health care delivery theme). Time limitations caused stress to both doctors and patients through regularly running behind time. In addition, participants mentioned patients changing GPs to see a doctor they perceived as giving patients more time could be stressful as this behaviour was noted to have wider ramifications:

*Then of course you make a rod for your own back because I think by giving people more time and addressing more problems than you should, word gets around, people change to you because a friend recommends you. I've even had people change from doctors within the practice saying that they don't like Dr. So-and-So because he's always in such a hurry and so brusque and efficient, and your heart just sinks because you think, well yes, I will try and do a good job and give more time, but that comes at a cost to me and to my other patients, so you run later and later. (Participant 9 GP)*

#### Inadequacy of single disease guidelines

Participants expressed concern about the use of clinical practice guidelines for people with multimorbidity, which are generally developed for single clinical conditions. They were felt as adding to the complexity of managing multimorbidity through the difficulty in applying multiple guidelines in a single patient:

*Whereas if you have got one person with diabetes, it’s fairly straightforward to follow the guidelines. People with multiple conditions, there are guidelines for each of them, and it’s impossible…, it’s not beneficial to the patient to stick to 4 guidelines for 4 conditions (Participant 7 GP).*

#### Addressing clinical decision making in multimorbidity: “satisficing” and relational continuity of care

Participants reported two main strategies they used to address the problems of clinical decision making with a patient with multimorbidity: “satisficing” and relational continuity of care.

The first strategy, used in a single consultation, was that of squaring the need to deliver optimal disease management and patient-centred care in a time-limited consultation. This strategy, termed “satisficing”, can be defined as “settling for chronic disease management that was satisfactory and sufficient, given the particular circumstances of that patient” [27]. One commonly used approach was that of relaxing treatment targets to below those recommended by clinical guidelines:

*I think, not perfectly managed, but managed well enough within that person’s individual parameters. (Participant 6 GP)*

Another approach was to negotiate a compromise with the patient over which aspects of a recommended management plan needed to be adhered to:

*I think it comes down to agreed management plan and I think that's really the point. There's some things you're going to agree and some things you don't. I think that's probably the point. It's an agreed management system, which is again why the imposition of targets and the imposition of a certain way of doing chronic disease management doesn't work. (Participant 4 GP)*

When the patient’s multiple conditions were viewed as stable the emphasis from GPs was that this stability should be maintained, rather than constantly strive to implement guideline recommendations:

*So, although, in an ideal world I would say he should lose 20 kg and be completely pain-free from his back problems and not take any painkillers and not take this group of several medications that he's on, I think it's not a bad situation in that we're managing it and it's stable and relatively well-managed. (Participant 9 GP)*

The second strategy participants used was to harness the longitudinal nature of the patient-primary care practitioner encounter through the provision of relational (personal) continuity of care (“an ongoing therapeutic relationship between a patient and one or more providers”) [28]. This was the predominant approach used by participants and allowed GPs to both establish an ongoing personal relationship with patients and to ensure problems identified but not addressed in a first consultation were dealt with at subsequent consultations. Thus, GPs would address the time limitations of the single consultation by negotiating a subsequent consultation, employing an “additive-sequential” [29] approach to clinical decision making:

*Sometimes if they've got a whole list of things you have to just sort of divide the list up and say, "Look, we'll do this today and maybe we can, we need to do something about these things, but then you can come back and we'll do the other thing," (Participant 2 GP)*

Over the course of a year, 3 or 4 follow up appointments with the same GP allowed these ongoing issues to be addressed sequentially:

*… with the people with multimorbidity you see frequently it’s not just one 15 min time slot, it’s just carrying on from where you left off last time. You build and build and build on that. In the year, you’ve had an hour and probably more. (Participant 6 GP)*

The use of relational continuity of care as a tool to address multimorbidity was not limited to GPs. PCNs stated that they had longer consultation time with patients than GPs which provided space to get to know patients and helped initiate and reinforce self-management behaviour in the long term management of a patient’s condition:

*I work kind of half hour appointment. I aim for the first 10 min to be the patient introduction … you know let them talk about whatever they would like to talk about and then I would bring it round to the last 20 min to hone in on specifically what I would like, tied in with what they would like. But I do have a lot of time with my patients and that's [how] you know I get to know them, what they've been up to, has anything happened in their lives recently, what are they worried about, you know, “how is the pet?. So we cover the social aspect and we just gently move around to the diabetes side of things (Participant 8 PCN)*

Like GPs, one way they achieved this was to utilise multiple consultations to allow this advice to be delivered sequentially over time:

*It's repeating the message, but repeating it in different ways. Sometimes I'll dwell on the medication management, other times I might dwell on their lab results, another time … well I always talk about the lifestyle really. Just putting the stress on different things and finding out what clicks to people. Trying to find something that's sort of like, you know what it’s like yourself. People can tell you the same thing but in one day someone will say it slightly different and you think oh! (Participant 3 PCN)*

Although relational continuity of care was the predominant approach utilised, one participant noted that patients in their practice were increasingly seeing several GPs in their practice as the GPs were all part-time and s/he that noted management continuity of care (“a consistent and coherent approach to the management of a health condition that is responsive to a patient’s changing needs”) [28] was becoming more important for their patients with multimorbidity.

### Health care delivery

#### Primary care funding model

Participants felt that the current mixed capitation/co-payment model of NZ primary care was a barrier to the delivery care for patients with multimorbidity. The current level of capitation funding was considered too low and the 50/50 split between capitation and co-payments created a particular challenge for NZ primary care:

*I think New Zealand is in many ways the most difficult [setting to practise GP] because you have two customers scrapping for the same amount of time thinking that they're your exclusive customer. You've got the funding from the ministry of health through the PHOs … [who] are not going to pay you if you don't tick [their] boxes. You've got your patient with their A3 list, and both of them want 20 min at least of the 15 min appointment. There's 40 min. Two customers fighting for the same time window. (Participant 16 GP)*

The need for patient co-payment was also identified by participants as a specific barrier to the employment of the “additive-sequential” approach to clinical decision making described above:

*They [Patients] will say, “Oh, this, this, and this.” I’ll say, “Well look, we can deal with this and deal with this, but the other, that sounds really important and I don’t want to dismiss it. You will need to make another appointment to come back.” That’s very difficult, because I’m very aware that we charge [NZD] $39 for a consultation. I am very aware that a significant number of people in our area, that’s a big portion of the money that they’re getting that week. It’s not easy. It’s not easy to do that. (Participant 10 GP)*

#### Use of *Care Plus*

Participants reported that *Care Plus* funding was used flexibly and in three broad ways by general practices to provide care for patients with multimorbidity. The funding could be used to subsidise GP visits only; visits with both GP and PCN; and visits to the PCN only. Within each of these approaches various ways of delivering consults with patients were described. The predominant model described was one of having one extended review appointment with a PCN (either a practice nurse or nurse practitioner) and then three subsidised appointments with the GP:

*Once they've registered with a nurse they then are entitled to three appointments with the GP for [NZD] $15.50 and they can use that as they choose. It has to be about their medical problem. (Participant 8 PCN)*

One participant described their general practice utilising a specific PCN-led *Care Plus* scheme in which relational continuity of care was encouraged:

*We've identified people in our practice who qualify under the Care Plus funding scheme or the High User Health Card, to have regular input free of charge to them. We use that money, we don't charge our patients and they get two hours of free nursing time a year. Usually in four half an hour visits, but we can tailor it to the individual needs. And they are assigned a specific nurse. We have a care plan screening questionnaire, a good assessment of them, where they're at and what their needs are and what help they've got. And the nurse assigned to that particular patient, the idea is that we build a relationship with them and if they have a hospital admission or their spouse has a hospital admission or one of them is ill, or the circumstances change, or just to support people better. (Participant 3 PCN)*

This model, however, was not typical and other participants described resistance from individual GPs for shifting regular (“three monthly”) reviews from GPs to PCNs for this group of patients.

There was no consensus among participants as to whether *Care Plus* had actually improved care for patients with multimorbidity. Two problems were identified. Firstly, it was seen as an “add on” capitation funding stream to subsidise a limited number (4) of consultations in “high needs” patients. In itself it was considered inadequate to allow a reorganisation of how NZ general practice delivers chronic disease management:

*What we've been trying to do for years is actually use the Care Plus funding in some way, which is actually quite difficult, as you're probably aware …. rather than [it] just be … an extra on top of people's care, to actually to use it to fund chronic disease management per se and to pull that in as a funding element for a new system … But it didn't work, A) because that's an enormous task, and we didn't have the resources and the ability to do that, B) there was no direct funding for that [chronic disease management] (Participant 4 GP)*

Secondly, participants considered that the *Care Plus* eligibility criteria meant that not all patients who met the criteria had complex health needs. It was not consistently delivered to patients with complex health needs (e.g., multiple long term conditions and polypharmacy) as defined in Ministry of Health guidance: [17]

*Care Plus is a pretty crude tool. You only need two long-term health conditions. It could be hypothyroidism and hypertension, you know, pretty straightforward conditions, really, so you get these people who are basically well, coming in every three months for their pills, and then people with eight conditions, who really need it. (Participant 10 GP)*

Participants also considered that some patients simply used *Care Plus* as a way to get their individual quota of subsidised annual appointments irrespective of clinical need to consult their GP more frequently. In other words, Care Plus was seen as a barrier to persuading patients to come back for another appointment, where a consultation had proven insufficient, because patients knew they had another subsidised appointment scheduled in three months’ time:

*For example, if I say, "Come back in two weeks to get your blood pressure checked," it's quite likely that they will just wait until the next routine visit, when their pills are running out, and then once again they'll use a Care Plus visit and get it cheaper. (Participant 9 GP)*

#### Fragmentation of health care provision

Fragmentation of health care was identified by participants as occurring both within NZ general practice and across the primary/secondary care interface. Within the primary care team participants noted that there had been a development of PCN-led single disease management clinics however this had to date not addressed multimorbidity:

*We had a blood pressure clinic and you [practice nurse] did your blood pressure. The respiratory clinic, and you did your respiratory, and so on. You only did a little bit of this, that, and the other. You didn't see the whole picture. (Participant 13 PCN)*

In order to develop a general practice team-based patient-centric model of care for people with multimorbidity participants noted that there was a need to fully implement chronic disease management models in primary care, something which required a culture shift:

*I think the concept of chronic disease management is a laudable prospect that should be delivered in primary care should be supported. I think rolling more of those ancillary services that are really designed for chronic disease management … needs to come into that area [primary care] … we need a philosophical shift as well..” (Participant 4 GP)*

*I think it's actually early days in the whole scheme of it [chronic disease management] - at the moment [we are] trying to change the culture of the separate conditions. (Participant 13 PCN)*

Participants described fragmentation across primary and secondary care as systemic and pervasive and the result of a model that was outdated in relation to the context it applied in (i.e., an ageing population with increasing prevalence of long-term conditions/chronic disease and multimorbidity). This fragmentation was seen as institutional, caused by the disconnect between primary and secondary care:

*The trouble is at the moment there’s no viable model about sharing which would allow us to proceed and obviously because we’ve got two different kinds of systems and not really kind of integrated so it’s a difficult one. (…) The trouble is that specialist medicine doesn’t appreciate a shared model of care really. (Participant 12 GP)*

Participants were keen to see secondary care and primary care working more closely together and emphasised the need for better communication. Reference was made to recent initiatives such as shared computer records, electronic patient referral systems and telehealth as promoting better integration, but no specific examples were given as to where these initiatives had improved the care of patients with multimorbidity in the NZ health system either nationally or locally.

## Discussion

### Statement of principal findings

This is the first NZ-based study to specifically explore primary care professional accounts of managing multimorbidity and its impact on clinical decision making and health delivery. Primary care professionals (GPs and PCNs) encountered challenges in providing care to patients with multimorbidity with respect to both clinical decision making and health care delivery. Clinical decision making occurred in time-limited consultations where the challenges of complexity and inadequacy of single disease guidelines were managed through the use of “satisficing” (care deemed satisfactory and sufficient for a given patient) and sequential consultations utilising relational continuity of care. The NZ primary care co-payment funding model was seen as a barrier to the delivery of care as it discourages sequential consultations, a problem only partially addressed through the use of the additional capitation based funding stream of *Care Plus*. Fragmentation of care also occurred within general practice in relation to the distribution of care between health professional roles, as well as across the primary/secondary care interface. This latter fragmentation was due in part to a lack of system supported relationships between primary and secondary care and good communication processes (e.g., shared information systems).

### Strengths and limitations

This qualitative interview study utilised purposive sampling to enable a maximum variation sample in terms of characteristics of participants (sex, ethnicity and years in practice) and general practice demographics (practice size, location and level of socioeconomic deprivation). The choice of individual interviews was appropriate as we wished to focus both on individual clinical decision making and health care delivery issues: an approach utilised in similar research studies in the UK [29, 30] and Ireland [27]. We chose to focus on one NZ health region (Otago) as this work was intended to inform Southern Health Region health service development for people with multimorbidity and it was also a requirement of the research funder that the work was conducted in this region. The inclusion of both GPs and PCNs meant that we were able to gain insight into the varying ways their roles are enacted in different practice settings to manage multi-morbidity. We were able to recruit a maximum variation GP sample and achieve data saturation (no new emergent themes) for the GP interviews. Furthermore, the interviews and emergent themes were subject to ongoing discussion and refinement within the research team and we consider our results are conceptually (theoretically) generalizable [31].

It is accepted, however, that those who participated are likely to have a greater interest in the subject matter than those who declined to participate. In addition, we were not able to recruit any primary care professionals who were Māori or Pasifika. More generally, the choice to conduct this study in one defined geographical region limits the ability to fully explore potential variation in health care delivery for this group across NZ. While we had not set a fixed number of planned interviews for both GPs and PCN interviews we had difficulty recruiting PCNs and only managed to interview 4 in total. We do not consider we were able to fully explore PCN accounts of managing multimorbidity and further NZ research is required here. It is also important to note that in conducting interviews we collected situated accounts [31] from health care professionals, thus we have described what people say they do, not what they necessarily did. Finally, the accounts are from the health care professionals only and as such provide no information regarding the patient’s perspective of care when managing multimorbidity. In other health systems, a key finding in a recent review of the patient experience literature on multimorbidity is lack of holistic care [32].

### Comparison with existing literature

There is a limited NZ research literature on managing multimorbidity in primary care [33–35] and none addresses the specific aims of this study. The findings reported in the *clinical decision making* theme are, however, consistent with the challenges of managing multimorbidity reported by general practitioners [13, 27] and PCNs [30, 36] working in similar first contact primary care health systems. Specifically, a meta-ethnography (qualitative synthesis) of the research literature up to 2012 identified three areas of difficulty which are also described here: inadequacy of guidelines and evidence-based medicine, challenges in delivering patient-centred care and barriers to shared decision making [13]. In our study we specifically report an inadequacy of single disease guidelines theme and consider challenges relating to shared decision making across several of the other reported themes, notably the “complexity” theme. The concept of “satisficing”, which was one of the two main strategies participants used to address the problems of clinical decision making was first used in the multimorbidity literature by Sinnott and colleagues in their qualitative study of Irish GPs and prescribing in multimorbidity [27]. In terms of the second strategy, relational continuity of care (“an ongoing therapeutic relationship between a patient and one or more providers”) [28] has also been identified as being perhaps the most important facilitator of care in multimorbidity in primary care. Relational continuity of care allows primary care practitioners to “foster trust, anticipate preferences and empower their patients over time” [13].

It is in the *health care delivery* theme that NZ health system specific barriers and facilitators are reported. Thus while the NZ primary care professionals, like their UK equivalent, used the “additive-sequential” clinical decision making approach [29] they faced an additional issue not encountered in the UK: the NZ co-payment model. This co-payment model means that patients encounter a financial barrier to see their primary care practitioner for repeated consultations. Inability to access primary care in NZ due to financial barriers is widely reported in the NZ health literature. For example, in a 2009 NZ national survey [37] 15.5% of respondents reported that they had deferred seeing their doctor/s at least once during the preceding 12 months, because they could not afford the cost of a visit and the presence of more than two co-morbid diseases was independently associated with increased odds of deferring doctors' visits. This finding is also consistent with a recent Commonwealth Fund Survey, where NZ was rated third worst (behind Switzerland and US) of 11 countries for adults going without needed health care because of costs [38].

Internationally, the most common model of chronic disease/long term condition management that underpins models of multimorbidity care, including NZ’s *Care Plus* [17] is Wagner’s chronic care model [39–41]. One key aspect of this model, the core approach of NZ’s *Care Plus* - that of enabling delivery of extended appointments to people with multimorbidity – has also been used in other health systems [41] and is a key component of an ongoing Scottish multimorbidity complex intervention evaluation [42]. NZ *Care Plus* has, however, not been the subject of an independent evaluation of its effectiveness or cost effectiveness in spite of it being in operation since 2004. To date, its evaluation has been local (North Island PHO) and has focussed on exploring primary care practitioners’ perceptions [43]. Our final finding, that of fragmentation of care, is also reported in the international literature [13]. The NZ health system, including the Southern Health region, [44] compares unfavourably with other health systems in terms of the degree of care fragmentation [11, 45].

### Implications for clinical practice, health policy and research

The NZ GPs and PCNs in this study utilised a clinical decision making approach for patients with multimorbidity consistent with that used in other health systems with strong primary care: [10] that of “satisficing” [27] and relational continuity of care (delivered through an “additive-sequential” [29] model of consecutive consultations). It therefore adds to the evidence base on how primary care practitioners make clinical decisions for this group of patients and also suggests that recent evidence-based guidance on the clinical management of multimorbidity developed by the National Institute for Health and Care Excellence (NICE) for the UK National Health Service is likely to be generalizable to, and therefore implementable in, NZ primary care [46].

This study has also identified three key barriers to the delivery of health care to patients with multimorbidity in NZ: the primary care funding model, the variable interpretation and implementation of the *Care Plus* scheme by NZ general practices and the fragmentation of health care within general practices and across the health system. While all three elements will need to be addressed by NZ health policy makers if NZ is to have an equitable and integrated health care system there is a clear opportunity to review and revise *Care Plus* so it better meets its stated objectives in relation to long term conditions [17]. A key finding from this study is that simply focussing on “adding on” additional capitation for patients with multimorbidity without addressing the need to redesign primary and secondary health care delivery around their health needs [9] may lead to little or no health gain. One way forward would be to develop a more structured approach to *Care Plus* that specifically ensures it addresses all components of chronic disease management [39] – key elements will be a clear operational definition of who is eligible for the scheme, sufficient funding of extended and review consultations (so enabling relational continuity of care), training and support for general practices to deliver a structured approach to identifying patients’ priorities for care and supporting patient self management. It will also be important to develop a model that is responsive to the range of health and social care needs of people with multimorbidity and recognises the high prevalence of multimorbidity in the population, as opposed to only focussing on the smaller group of patients with complex health care needs (e.g., frail elderly at risk of repeated readmission to hospital) [47]. Finally, there is an important need for NZ health research funders to commission independent research to evaluate at a national level the effectiveness and cost effectiveness of this revised *Care Plus* model of care delivery compared to usual care – as has been recommended [46] and commissioned [48] in other comparable health systems.

## Conclusions

This study highlights specific New Zealand barriers to the delivery of primary care to patients living with multimorbidity: primary care professionals encounter challenges in providing care to patients with multimorbidity with respect to both clinical decision making and health care delivery. A key finding is that the New Zealand primary care co-payment funding model is seen as a barrier to the delivery of care as it discourages sequential consultations, a problem only partially addressed through the use of the additional capitation based funding stream of *Care Plus*. There is a need to develop, implement and nationally evaluate a revised version of *Care Plus* that takes account of these barriers.

## Additional files

Additional file 1: Interview Topic Guide. The semi-structured interview topic guide questions and probes. (PDF 261 kb)

Additional file 2: COREQ-32 reporting checklist. An assessment of the study reporting against the domains of the COREQ-32 reporting checklist for interviews and focus groups. (PDF 208 kb)

## Abbreviations

DHB: District health board
GP: General practitioner
HUHC: High user health card
NZ: New Zealand
PCN: Primary care nurse
PHO: Primary Health Organisation

## References

[1] Valderas JM, Starfield B, Sibbald B, Salisbury C, Roland M (2009). Defining comorbidity: implications for understanding health and health services. Ann Fam Med.

[2] Mangin D, Heath I, Jamoulle M (2012). Beyond diagnosis: rising to the multimorbidity challenge. BMJ.

[3] Goodwin N, Dixon A, Anderson GF, Wodchis W (2014). Providing integrated care for older people with complex needs Lessons from seven international case studies.

[4] Mercer SW, Smith SM, Wyke S, O'Dowd T, Watt GC (2009). Multimorbidity in primary care: developing the research agenda. Fam Pract.

[5] Violan C, Foguet-Boreu Q, Flores-Mateo G, Salisbury C, Blom J, Freitag M, Glynn L, Muth C, Valderas JM (2014). Prevalence, determinants and patterns of multimorbidity in primary care: a systematic review of observational studies. PLoS One.

[6] Barnett K, Mercer SW, Norbury M, Watt G, Wyke S, Guthrie B (2012). Epidemiology of multimorbidity and implications for health care, research, and medical education: a cross-sectional study. Lancet.

[7] Wolff JL, Starfield B, Anderson G (2002). Prevalence, expenditures, and complications of multiple chronic conditions in the elderly. Arch Intern Med.

[8] Salisbury C, Johnson L, Purdy S, Valderas JM, Montgomery AA (2011). Epidemiology and impact of multimorbidity in primary care: a retrospective cohort study. Br J Gen Pract.

[9] Smith SM, Wallace E, O'Dowd T, Fortin M (2016). Interventions for improving outcomes in patients with multimorbidity in primary care and community settings. Cochrane Database Syst Rev.

[10] Starfield B, Shi L, Macinko J (2005). Contribution of primary care to health systems and health. Milbank Q.

[11] Mays N: Reorienting the New Zealand health care system to meet the challenge of long-term conditions in a fiscally constrained environment. In: Paper prepared for New Zealand Treasury Long-term Fiscal External Panel, November 2012, and Chair of Public Finance, Victoria University of Wellington and New Zealand Treasury conference, Affording our Future, Wellington, 10-11 December 2013. (http://www.hiirc.org.nz/page/37816). Accessed 22 February 2017.

[12] McWhinney IR, Freeman T (2009). Textbook of Family Medicine.

[13] Sinnott C, Mc Hugh S, Browne J, Bradley C (2013). GPs' perspectives on the management of patients with multimorbidity: systematic review and synthesis of qualitative research. BMJ Open.

[14] Gauld R, Mossialos E, Wenzl M, Osborn R, Sarnak D (2016). The New Zealand Health Care System. 2015 International Profiles of Health Care Systems.

[15] Ministry of Health (MoH) (2016). Very Low Cost Access Scheme (VLCA).

[16] Carryer J, Doolan-Noble F, Gauld R, Budge C (2014). New Zealand patients' perceptions of chronic care delivery. J Integr Care.

[17] Ministry of Health (2004). Care Plus: An Overview.

[18] CBG Health Research Limited (2003). Care Plus Formative Report Prepared for Ministry of Health.

[19] CBG Health Research Limited (2004). Care Plus Process Report Prepared for Ministry of Health.

[20] Ministry of Health (2015). Eligibility for Care Plus.

[21] Gauld R (2014). What should governance for integrated care look like? New Zealand's alliances provide some pointers. Med J Aust.

[22] Southern District Health Board (2015). Southern Strategic Health Plan Piki te Ora.

[23] 2013 New Zealand Census QuickStats about a place: Otago Region (http://www.stats.govt.nz/Census/2013-census/profile-and-summary-reports). Accessed 22 February 2017.

[24] Braun V, Clarke V (2006). Using thematic analysis in psychology. Qual Res Psychol.

[25] Glaser BG (1965). The constant comparative method of analysis. Soc Probl.

[26] Tong A, Sainsbury P, Craig J (2007). Consolidated criteria for reporting qualitative research (COREQ): a 32-item checklist for interviews and focus groups. Int J Qual Health Care.

[27] Sinnott C, Hugh SM, Boyce MB, Bradley CP (2015). What to give the patient who has everything? A qualitative study of prescribing for multimorbidity in primary care. Br J Gen Pract.

[28] Haggerty JL, Reid RJ, Freeman GK, Starfield BH, Adair CE, McKendry R (2003). Continuity of care: a multidisciplinary review. BMJ.

[29] Bower P, Macdonald W, Harkness E, Gask L, Kendrick T, Valderas JM, Dickens C, Blakeman T, Sibbald B (2011). Multimorbidity, service organization and clinical decision making in primary care: a qualitative study. Fam Pract.

[30] O'Brien R, Wyke S, Guthrie B, Watt G, Mercer S (2011). An 'endless struggle': a qualitative study of general practitioners' and practice nurses' experiences of managing multimorbidity in socio-economically deprived areas of Scotland. Chronic Illn.

[31] Murphy E, Dingwall R, Greatbatch D, Parker S, Watson P (1998). Qualitative research methods in health technology assessment: a review of the literature. Health Technol Assess.

[32] Van der Aa M, Van den Broeke JR, Stronks K, Ploch T (2017). Patients with multimorbidity and their experiences with the healthcare process: a scoping review. J Comorbidity.

[33] Doolan-Noble F, Gauld R, Waters DL (2015). Are nurses more likely to report providing care plans for chronic disease patients than doctors? Findings from a New Zealand study. Chronic Illn.

[34] Ailabouni NJ, Nishtala PS, Mangin D, Tordoff JM (2016). General practitioners' insight into deprescribing for the multimorbid older individual: a qualitative study. Int J Clin Pract.

[35] McKinlay E, Graham S, Horrill P (2015). Culturally and linguistically diverse patients' views of multimorbidity and general practice care. J Prim Health Care.

[36] Jowsey T, Jeon YH, Dugdale P, Glasgow NJ, Kljakovic M, Usherwood T (2009). Challenges for co-morbid chronic illness care and policy in Australia: a qualitative study. Aust N Z Health Policy.

[37] Jatrana S, Crampton P (2009). Primary health care in New Zealand: who has access?. Health Policy.

[38] Osborn R, Squires D, Doty MM, Sarnak DO, Schneider EC (2016). In New Survey Of Eleven Countries, US Adults Still Struggle With Access To And Affordability Of Health Care. Health Aff.

[39] Wagner EH, Austin BT, Von Korff M (1996). Organizing care for patients with chronic illness. Milbank Q.

[40] Bodenheimer T, Wagner EH, Grumbach K (2002). Improving primary care for patients with chronic illness: the chronic care model, Part 2. JAMA.

[41] Stokes J, Man, MS., Bower, P., Guthrie, B., Mercer, C., Salisbury, C. : Models of Primary Care for Chronic Conditions and Multimorbidity: Comparing current approaches North American Primary Research Group (NAPCRG). Colorado Springs; 2016. (http://www.napcrg.org/Conferences/PastMeetingArchives/2016AnnualMeetingArchives). Accessed 22 February 2017.

[42] Mercer SW, Fitzpatrick B, Guthrie B, Fenwick E, Grieve E, Lawson K, Boyer N, McConnachie A, Lloyd SM, O'Brien R (2016). The CARE Plus study - a whole-system intervention to improve quality of life of primary care patients with multimorbidity in areas of high socioeconomic deprivation: exploratory cluster randomised controlled trial and cost-utility analysis. BMC Med.

[43] Eggleton K, Kenealy T (2009). What makes Care Plus effective in a provincial Primary Health Organisation? Perceptions of primary care workers. J Prim Health Care.

[44] Doolan-Noble F, Gauld R, Walters DL, de la Barra SL (2015). New Zealand health professional and patient perceptions of chronic illness care. Eur J Person Centered Healthc.

[45] Ham C (2010). The ten characteristics of the high-performing chronic care system. Health Econ Policy Law.

[46] National Guidelines Centre (2016). Multimorbidity: clinical assessment and management [NICE Clinical Guideline NG56].

[47] Ministry of Health (2016). Self-management Support for People with Long-term Conditions.

[48] Mann C, Shaw A, Guthrie B, Wye L, Man MS, Hollinghurst S, Brookes S, Bower P, Mercer S, Salisbury C (2016). Protocol for a process evaluation of a cluster randomised controlled trial to improve management of multimorbidity in general practice: the 3D study. BMJ Open.

[49] Gauld R (2013). Questions about New Zealand's health system in 2013, its 75th anniversary year. N Z Med J.

