# Supplementary material for: Multimorbidity, clinical decision making and health care delivery in New Zealand Primary care: a qualitative study
Source: BMC Fam Pract. 2017 Apr 5;18:51. doi: 10.1186/s12875-017-0622-4 (PMC5382371; doi:10.1186/s12875-017-0622-4)
Supplement: Supplementary file 1 — Interview Topic Guide. The semi-structured interview topic guide questions and probes. (PDF 261 kb) [file 12875_2017_622_MOESM1_ESM.pdf]

Interview Intro & Suggested Questioning Framework

"CONSENT PROCESS" - Before we begin, do you have any further questions

The following questions are examples rather than a set of specific questions. Questions in bold are the main questions. Those in grey are possible prompting questions. Interviews did not necessarily flow in a linear manner as per the question guide.

**Question**
Could you tell me a bit about yourself and your practice setting?<br>How long have you been at this practice?<br>How long have you been qualified?<br>Have you completed any other qualifications on top of your medical/nursing degree?<br>What is your list size? How does your practice work?
Could you please describe the management of people with chronic conditions within your practice setting (single disease)?<br>GP/Nurse responsibilities?<br>Multi-visit management and Careplus?<br>Link to secondary/tertiary care?<br>Link to community support/management?<br>Frustrations? Rewards?
Could you tell me how you define multimorbidity?
What is the impact of multimorbidity on your practice setting?<br>Growing numbers? Growing complexity?<br>Is the management of patients with multimorbidity within your practice a burden, and if so why? (i.e. time, costs, etc.).<br>Do you find the same kinds of issues arise for all multimorbidity patients or is there a high degree of variability between management of patients with multimorbidity?<br>How does the administrative management of patients with multimorbidity differ from those without?
Could you describe a case of managing multimorbidity (anonymously) – what stands out for you in that management that is different from other patients?<br>What are the factors contributing to good management?<br>What are the factors contributing to poor management?<br>You mentioned XXX as working well, do you think this could be supported in other cases?<br>You mentioned XXX as working poorly, how do you think this could be changed?
Do you find anything particularly frustrating/difficult or rewarding managing multimorbidity?<br>You mentioned that you found XXX frustrating, do you think this could be resolved in anyway?<br>You mentioned that you found XXX rewarding is this the case for all your patients with multimorbidity, and if not, do you see a way of making this the case?
If you could make a recommendation/suggestion regarding changing the way in which care is provided to those patients with multimorbidity, what would it be?<br>Explore patient factors in relation to these recommendations for larger implications
