# Supplementary material for: Multimorbidity, clinical decision making and health care delivery in New Zealand Primary care: a qualitative study
Source: BMC Fam Pract. 2017 Apr 5;18:51. doi: 10.1186/s12875-017-0622-4 (PMC5382371; doi:10.1186/s12875-017-0622-4)
Supplement: Supplementary file 2 — COREQ-32 reporting checklist. An assessment of the study reporting against the domains of the COREQ-32 reporting checklist for interviews and focus groups. (PDF 208 kb) [file 12875_2017_622_MOESM2_ESM.pdf]

Domain 1:	Research Team and Reflexivity	
Personal Characteristics		
1.	Interviewer/facilitator	The Assistant Research Fellow (Emma Tumilty) conducted the interviews
2.	Credentials	Prof. Tim Stokes – MA MBChB MPH PhD (qualitative research methods)<br>Dr Fiona Doolan-Noble – MPHC PGDipPH RGN<br>Emma Tumilty – BA (Otago), PG Dip HealSci (Bioethics) (Otago), PhD (Bioethics – under examination) (Otago).<br>Prof. Robin Gauld - MA(Well) PhD(HK)
3.	Occupation	PhD candidate and Assistant Research Fellow
4.	Gender	Two male-identifying and two female identifying researchers (interviewer, female-identifying)
5.	Experience and training	Prof. Tim Stokes – experienced qualitative and mixed methods researcher with an associated publication track record of qualitative research studies<br>Dr Fiona Doolan-Noble – experienced qualitative and mixed methods researcher with an associated publication track record.<br>Emma Tumilty – previous experience undertaking qualitative interviews and analysing data in research projects prior to this study with an early career publication track record.<br>Prof. Robin Gauld – experienced health service and health policy researcher of international and national standing with an associated publication track record.
Relationship with participants		
6.	Relationship established	The assistant research fellow had no relationships with participants prior to the study, while the broader research team had varying relationships with the primary care community at large. It was felt the interviewer should be seen as an unbiased enquirer rather than a person with a particular stance or opinion about the topic.
7.	Participant knowledge of interviewer	The interviewer introduced herself to participants stating she was not a medical professional, etc. as well as describing the research team, its funding, the purpose of the project and answering any questions participants may have had about the project and those involved in it.
8.	Interviewer characteristics	The interviewer was not a medical professional, but aware of the medical system in New Zealand and had read the literature on multimorbidity prior

		to the begin of the project. She had previously worked as an research assistant on other projects involving qualitative interviews.
**Domain 2:**	**Study Design**	
Theoretical framework		
9.	Methodological orientation and theory	This project used an inductive method – data was analysed using the constant comparative approach.
Participant Selection		
10.	Sampling	Purposive and snowball sampling was used to recruit general practitioners, nurses, and nurse practitioners in the Otago region
11.	Method of approach	Various methods of approach were used, group emails were sent via a third party, invitations were added to newsletters, emails were sent to practices, and then those taking part or responding were also asked to pass on information to others. Direct emails were sent to practice managers, etc.
12.	Sample size	16
13.	Non-participation	There are 84 practices in the Otago region. A number of GPs and nurses did not respond to the invitation to participate, however no person responded directly to decline taking part, nor did anybody drop out once agreeing to take part.
Setting		
14.	Setting of data collection	Participants could determine whether the interview took place in their place of work or in a private meeting room arranged by the interviewer on the university campus. Participants were mixed in which of these options they chose.
15.	Presence of non-participants	No
16.	Description of sample	The sample was nine females and seven males. Four of the sample were nursing professionals while twelve were general practitioners. Eleven participants identified as NZ European, while five listed other ethnicities (including: Asian, Other European, African, etc.). The majority of participants had 20+ years of experience (9), with 3 have more than 10 years, 3 more than 5, and 1 less than 5.
Data collection		

17.	Interview guide	The interview guide was open, but listed a number of key questions and potential prompts. Interviews generally followed a similar approach although questions themselves varied depending on the participant.
18.	Repeat interviews	No
19.	Audio/visual recording	All interviews were audio-recorded and later transcribed.
20.	Field notes	Yes, the interviewer wrote brief field notes after each interview regarding the nature of the interview and her perceptions of the participants responses, etc.
21.	Duration	Interview times varied but were generally around 60mins (shortest 40mins, longest 85 mins)
22.	Data saturation	Data saturation was discussed within the research team as the interviewer undertook interviews and open-coded them in nVivo
23.	Transcripts returned	No
**Domain 3:**	**Analysis and Findings**	
Data Analysis		
24.	Number of data coders	The interviewer coded the data and described codes and these were discussed with two other members of the interview team on a regular basis
25.	Description of the coding tree	Codes had tags and description within the software except for where they were self-explanatory
26.	Derivation of themes	Themes were identified from the codes in team discussion
27.	Software	nVivo 11 was used to open code the data, describe codes and mark relationships
28.	Participant checking	No
Reporting		
29.	Quotations presented	Yes quotations are presented and identified in a manner protecting participants confidentiality
30.	Data and findings consistent	Consistency between the data and the findings exists and is largely consistent with pre-existing literature in other locations (internationally).
31.	Clarity of major themes	Yes, major themes are clearly identified
32.	Clarity of minor themes	Yes, minor themes are clearly identified and related to major themes.
